# An Improved Alignment Method for the Strapdown Inertial Navigation System (SINS)

**DOI:** 10.3390/s16050621

**Published:** 2016-04-29

**Authors:** Meng Liu, Yanbin Gao, Guangchun Li, Xingxing Guang, Shutong Li

**Affiliations:** College of Automation, Harbin Engineering University, Harbin 150001, China; liumeng_0304@163.com (M.L.); gaoyanbin@hrbeu.edu.cn (Y.G.); guangxingxing1008@163.com (X.G.); lishutongst@163.com (S.L.)

**Keywords:** polar alignment, pseudo-frame, decoupling, Kalman filter

## Abstract

In this paper, an innovative inertial navigation system (INS) mechanization and the associated Kalman filter (KF) are developed to implement a fine alignment for the strapdown INS (SINS) on stationary base. The improved mechanization is established in the pseudo-geographic frame, which is rebuilt based on the initial position. The new mechanization eliminates the effects of linear movement errors on the heading by decoupling. Compared with the traditional local-level mechanization, it has more advantages. The proposed algorithm requires lower coarse alignment accuracy in both the open-loop and closed-loop KFs and hence can improve the system reliability and decrease the total alignment time. Moreover, for the closed-loop KF, it can decrease oscillation caused by the system errors and improve the closed-loop system stability. In addition, the proposed algorithm can also be applied to polar alignment. The performance of the proposed algorithm is verified by both simulations and experiments and the results exhibit the superior performance of the proposed approach.

## 1. Introduction

The inertial navigation system (INS) has been widely used for the positioning and navigation of vehicles because it has particular advantages, such as high autonomy, concealment, continuum, insusceptible climate, and the successive supply of position, velocity, and attitude (PVA) information. strapdown INS (SINS), which has the advantages of simple structure, small size, and convenient maintenance compared with plat INS (PINS), is especially popular [1,2]. However, an accurate alignment must be required before the start of navigation. Otherwise, it will directly degrade navigation accuracy [3,4,5]. The purpose of the SINS’ initial alignment is to obtain the exact coordinate transformation matrix from the body frame to the navigation frame. Typically, this process consists of a coarse alignment and a fine alignment [6,7]. The objective of a coarse alignment is to provide a good condition for a fine alignment, where the misalignment angles are approximately estimated to a few degrees. In the fine alignment stage, the small misalignment angles are computed accurately and the precise initial transformation matrix is then formulated [8].

For a fine alignment, the Kalman filter (KF) is one of the most widely used methods due to its simplicity, optimality, tractability, and robustness [9,10]. Usually, the application of a KF in alignment can be categorized into two types: open-loop and closed-loop [11,12]. The estimation of SINS errors does not interfere with the operation of the SINS and is only subtracted from the output of the SINS in an open-loop system, whereas the estimation of the SINS errors is fed back into the strapdown computation processor in a closed-loop system. Therefore, one major drawback of an open-loop approach is that the system errors will be propagated (not courted to null). The large system errors may result in declining alignment accuracy or even lead to filter’s divergence. In a closed-loop system, the loop stability is also very sensitive to system errors, which cause the estimation oscillation that may lead to system divergence. For the fine alignment associated with the KF, the influences on the heading estimation will be more obvious because of its incomplete observation [13,14]. Furthermore, the worse the coarse alignment is, the larger the system errors are [6,15]. Therefore, the large coarse alignment errors will affect system stability and fine alignment performance. As a result, the KF alignment approach needs to exceedingly rely on the accuracy of coarse alignment, thus reducing system reliability. If using a traditional local-level mechanization for static alignment, the accuracy of the coarse alignment must be limited to a smaller degree. In other publications, an adaptive Kalman gain and larger initial values of the noise matrix are often used to decrease the influences of system errors on the closed loop [11,16,17]. However, the adaptive gain is difficult to predict and control. The larger initial values of the noise matrix can decrease the system errors’ influences but at the expensive of alignment performance. Consequently, in order to maintain the desired performance of fine alignment, more time is required to guarantee the accuracy of coarse alignment and hence an increase in alignment time.

On the other hand, with the development of the Northern Sea Route (NSR), the importance of polar navigation is increasing [18]. Due to the special geographical and electromagnetic conditions, the INS becomes a preferred choice for polar navigation [19]. Aiming at the polar algorithms of the SINS, the wander frame, the grid frame, and the transverse frame have been proposed for polar navigation [20,21,22]. However, polar alignment is still one of the toughest challenges for the SINS. The major problem arising in polar alignment is that the effects of linear movement errors (due to high latitude) are extremely vital for heading error propagation. In the literature, the wander mechanization can be applied to solve polar alignment. However, the wander approach cannot be widely used due to its cumbersome derivation and high computational complexity. Moreover, the alignment scheme also needs to be redesigned for the wander initial alignment, which is different from the conventional local-level alignment [23,24,25]. Therefore, the wander mechanization is an undesirable solution for polar alignment. As a result, a more simple mechanization must be required.

Aiming at the problems mentioned above, an innovative INS mechanization, which is established by reconstructing a pseudo-geographic frame with the initial position, is proposed to achieve a fine alignment for the SINS on stationary base, and the simulations and experiments are conducted to verify the performance of the proposed algorithm.

## 2. Reference Frames Definition

The different coordinate frames in this paper are defined as follows:

- i-frame: Earth-Centered Initially Fixed (ECIF) orthogonal reference frame.
- e-frame: Earth-Centered Earth-Fixed (ECEF) orthogonal reference frame.
- t-frame: Orthogonal reference frame aligned with East-North-Up (ENU) geographic frame.
- b-frame: body frame.
- n-frame: navigation frame.
- p-frame: Pseudo-Earth-Centered Earth-Fixed (PECEF) orthogonal reference frame obtained by two successive transformations from e-frame.
- $t_{p}$-frame: pseudo-orthogonal reference frame aligned with pseudo-East-North-Up $\left(E_{p}N_{p}U_{p}\right)$ pseudo-geographic frame.

## 3. The Analysis of Problem

In this section, the intention is to analyze the problem of the traditional static error model arising in the KF and polar alignment. Here, the local-level East-North-Up (ENU) frame is selected as the navigation frame. For the INS on stationary base, position and up velocity errors are usually ignored. From [22] (p. 253), the static velocity and attitude error model in the fine alignment state can be formulated as follows:

(1) $$\delta {\dot{V}}_{E}=2\Omega \sin L\delta V_{N}-\phi_{y}g+\nabla_{E}$$

(2) $$\delta {\dot{V}}_{N}=-2\Omega \sin L\delta V_{E}+\phi_{x}g+\nabla_{N}$$

(3) $${\dot{\phi }}_{x}=-\frac{\delta V_{N}}{R}+\phi_{y}\Omega \sin L-\phi_{z}\phi \cos L+\varepsilon_{E}$$

(4) $${\dot{\phi }}_{y}=\frac{\delta V_{E}}{R}-\phi_{x}\Omega \sin L+\varepsilon_{N}\;\mathrm{and}$$

(5) $${\dot{\phi }}_{z}=\frac{\delta V_{E}}{R}\tan L+\phi_{x}\Omega \cos L+\varepsilon_{U}$$

where Ω denotes the angular rate of Earth rotation; g denotes the local gravity; L and R denote the local latitude and the Earth radius respectively; and $\nabla_{E},\nabla_{N},\varepsilon_{E},\varepsilon_{N},\varepsilon_{U}$ denote the accelerometer zero-biases and the constant gyroscope drifts respectively.

From Equations (1) and (2), we can know that the worse the accuracy of the coarse alignment ($\phi_{x},\phi_{y},\phi_{z}$) is, the faster the level linear movement errors ($\delta V_{E},\delta V_{N}$) diverge. Then, the level linear movement errors will influence the misalignment angle’s spread in turn from Equations (3)–(5). Therefore, poor coarse alignment will result in large system errors. For the open-loop and closed-loop KFs, the large system errors may result in alignment accuracy or even lead to a filter’s divergence. As a result, the fine alignment system must exceedingly rely on coarse alignment. Furthermore, the fact that the heading error is not completely observable is widely accepted. Therefore, the influence of the level linear movement errors on heading error is more serious. On the other hand, from Equation (5), we can see that the propagation of the heading error is proportional to not only the linear movement error ($\delta V_{E}$) but also tangent function of latitude (tanL). Therefore, a small linear movement error may cause very large heading error in a high-latitude region. This is also one of the major difficulties for alignment in a high-latitude region, including polar alignment. Therefore, a decoupling mechanization between the linear movement errors and the heading error without much additional complexity is expected to be achieved.

## 4. The Principle of the Proposed Algorithm

### 4.1. The Definition of the Pseudo-Frame

**Figure 1:**
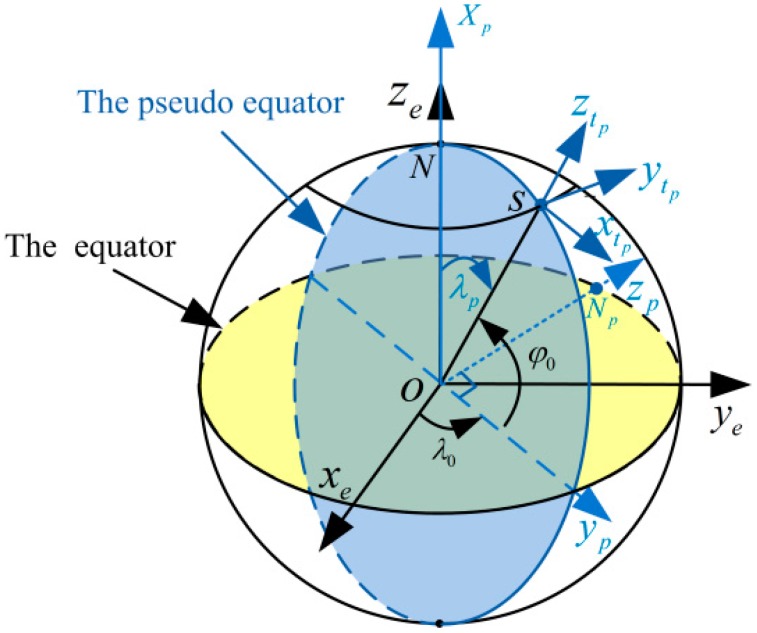
The pseudo-coordinate system.

In this paper, the decoupling mechanization is established in a pseudo-geographical frame. Suppose the Earth is spherical and the initial position (latitude and longitude) of the vehicle is ($L_{0},\lambda_{0}$) in e-frame. The p-frame ($o_{p}x_{p}y_{p}z_{p}$) can be obtained by two successive transformations with two Euler angles from the e-frame ($o_{e}x_{e}y_{e}z_{e}$), as shown in Figure 1. Additionally, the rotations are performed in the following order:

$$o_{e}x_{e}y_{e}z_{e}\overset{rotate\;around\;y_{e}\;axis\;}{\underset{-90^{\circ }}{\to }}o_{e}x_{e}^{\prime }y_{e}^{\prime }z_{e}^{\prime }\overset{rotate\;around\;x_{e}^{\prime }\;axis}{\underset{\lambda_{0}-90^{\circ }}{\to }}o_{p}x_{p}y_{p}z_{p}$$

According to the above description, the transformation matrix from e-frame to p-frame can be derived as Equation (6).

(6) $$C_{e}^{p}(\lambda_{0})=\left[\begin{array}{ccc} 0 & 0 & 1\\ \cos\lambda_{0} & \sin\lambda_{0} & 0\\ -\sin\lambda_{0} & \cos\lambda_{0} & 0 \end{array}\right]$$

As shown in Figure 1, $N_{p}$ denotes the pseudo-North Pole; the origin of the pseudo-Earth-Centered Earth-fixed frame ($o_{p}x_{p}y_{p}z_{p}$) is at the Earth’s mass center; the $o_{p}x_{p}$ axis points towards the North Pole, which is also known as pointing towards the pseudo-prime meridian; the $o_{p}y_{p}$ axis is pointing from the center of the Earth to the projection point of the carrier’s normal frame position S on the equator plane; the $o_{p}z_{p}$ axis is perpendicular to the $o_{p}y_{p}$ axis and conforms to the rules of the right-hand spiral in the equator plane, which is also known as pseudo-North Pole axis.

For the definition of the pseudo-coordinate system, it is similar to the normal coordinate system. $L_{p}$ and $\lambda_{p}$ represent pseudo-latitude and pseudo-longitude, respectively. Moreover, the $ox_{t_{p}}y_{t_{p}}z_{t_{p}}$ frame denotes $t_{p}$-frame in pseudo-Earth frame as shown in Figure 1. The origin of $ox_{t_{p}}y_{t_{p}}z_{t_{p}}$ frame is at the mass center of the vehicle; the $oz_{t_{p}}$ axis is normal to the local-level frame (denoted as $U_{p}$ axis); the $oy_{t_{p}}$ axis is pointing towards the pseudo-north direction (denoted as $N_{p}$ axis); and the $ox_{t_{p}}$ axis completes a right-handed system (denoted as $E_{p}$ axis).

From the above pseudo-frames definition, the pseudo-equator overlaps the normal longitude circle in the initial position. Therefore, the carrier’s initial position in pseudo-frame can be given as Equation (7).

(7) $$\left[\begin{array}{cc} L_{p0} & \lambda_{p0} \end{array}]=[\begin{array}{cc} 0^{\circ } & 90^{\circ }-L_{0} \end{array}\right]$$

Moreover, the transformation matrix from the t-frame to the $t_{p}$-frame can be expressed as Equation (8).

(8) $$C_{t}^{t_{p}}=C_{p}^{t_{p}}\cdot C_{e}^{p}(\lambda_{0})\cdot C_{t}^{e}$$

where $C_{t}^{e}={\left(C_{e}^{t}\right)}^{T}$; $C_{p}^{t_{p}}$ is a function of pseudo-latitude and -longitude; $C_{e}^{t}$ is a function of latitude and longitude; and $C_{p}^{t_{p}}$ and $C_{e}^{t}$ are shown in Equations (9) and (10), respectively.

(9) $$C_{p}^{t_{p}}=\left[\begin{array}{ccc} -\sin\lambda_{p} & \cos\lambda_{p} & 0\\ -\sin L_{p}\cos\lambda_{p} & -\sin L_{p}\sin\lambda_{p} & \cos L_{p}\\ \cos L_{p}\cos\lambda_{p} & \cos L_{p}\sin\gamma_{p} & \sin L_{p} \end{array}\right]$$

(10) $$C_{e}^{t}=\left[\begin{array}{ccc} -\sin\lambda & -\sin L\cos\lambda & \cos L\cos\lambda \\ \cos\lambda & -\sin L\sin\lambda & \cos L\sin\gamma \\ 0 & \cos L & \sin L \end{array}\right]$$

Substituting Equations (6), (7), (9) and (10) into Equation (8), the transformation matrix from the t-frame to the $t_{p}$-frame at initial position can be given as Equation (11).

(11) $$C_{t}^{t_{p}}(0)=\left[\begin{array}{ccc} 0 & -1 & 0\\ 1 & 0 & 0\\ 0 & 0 & 1 \end{array}\right]$$

From Equation (11), it can be obtained that the $t_{p}$-frame ($E_{p}N_{p}U_{p}$) overlaps the traditional South-East-Up (SEU) geographic frame at the initial position. As a result, the SINS alignment mechanization in $t_{p}$-frame will be easier to implement without much additional complexity.

### 4.2. The Mechanization of the SINS in Pseudo-Frame

In the pseudo-coordinate system, the $t_{p}$-frame is selected as the navigation frame (n-frame), and the SINS mechanization in the $t_{p}$-frame is similar to the one in the t-frame. Therefore, the rotation angular rate of the p-frame relative to the i-frame (due to the Earth spin angle rate) decomposed in n-frame can be written as Equation (12).

(12) $$\omega_{ip}^{n}={\left[\begin{array}{ccc} -\Omega \sin\lambda_{p} & -\Omega \sin L_{p}\cos\lambda_{p} & \Omega \cos L_{p}\cos\lambda_{p} \end{array}\right]}^{T}$$

where $L_{p}$ and $\lambda_{p}$ denote pseudo-latitude and pseudo-longitude in pseudo-Earth frame respectively. 

Moreover, the rotation angular rate of the local n-frame relative to the p-frame (due to vehicle movement over the Earth surface) decomposed in n-frame is expressed as Equation (13).

(13) $$\omega_{pn}^{n}={\left[\begin{array}{ccc} -\frac{V_{N_{p}}}{R} & \frac{V_{E_{p}}}{R} & \frac{V_{E_{p}}}{R}\tan L_{p} \end{array}\right]}^{T}$$

where $V_{E_{p}}$ and $V_{N_{p}}$ represent the level velocity vectors in $t_{p}$-frame. 

Considering Equations (12) and (13), the total rotation angular rate of the n-frame relative to the i-frame decomposed in n-frame can be given as Equation (14).

(14) $$\omega_{in}^{n}=\omega_{ip}^{n}+\omega_{pn}^{n}\;={\left[\begin{array}{ccc} -\Omega \sin\lambda_{p}-\frac{V_{N_{p}}}{R} & -\Omega \sin L_{p}\cos\lambda_{p}+\frac{V_{E_{p}}}{R} & \Omega \cos L_{p}\cos\lambda_{p}+\frac{V_{E_{p}}}{R}\tan L_{p} \end{array}\right]}^{T}$$

**Figure 2:**
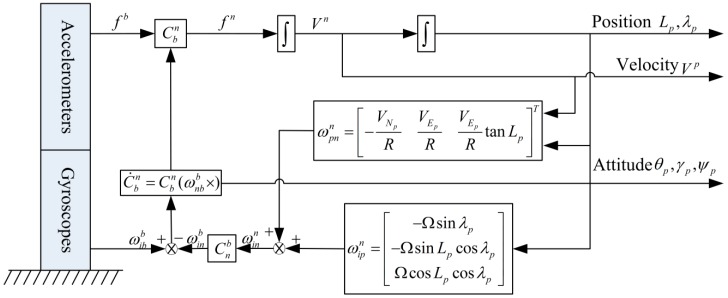
The strapdown inertial navigation system (SINS) mechanization in pseudo-geographic frame.

According to the above analysis, the SINS mechanization in the pseudo-geographic frame is given as shown in Figure 2.

According to the above mechanization in pseudo-geographic frame, the navigation equations can be written as Equations (15)–(18).

(15) $${\dot{C}}_{b}^{n}=C_{b}^{n}(\omega_{nb}^{b}\times )$$

(16) $${\dot{V}}^{n}=C_{b}^{n}f^{b}-(2\omega_{ip}^{n}+\omega_{pn}^{n})\times V^{n}+g^{n}$$

(17) $$\dot{\lambda }=\frac{V_{E_{p}}\sec L_{p}}{R}\;\mathrm{and}$$

(18) $${\dot{L}}_{p}=\frac{V_{N_{p}}}{R}\;$$

where $\omega_{nb}^{b}=\omega_{ib}^{b}-\omega_{in}^{b}=\omega_{ib}^{b}-C_{n}^{b}\omega_{in}^{n}$, $C_{n}^{b}={\left(C_{b}^{n}\right)}^{T}$; $\left(\omega_{nb}^{b}\times \right)$ denotes a skew-symmetric matrix of $\omega_{nb}^{b}$; $f^{b}$ denotes the specific force measured by accelerometer; $\omega_{ib}^{b}$ denotes the body angular rate measured by gyroscope; and $C_{b}^{n}$ denotes the attitude transformation matrix.

### 4.3. The Static Error Equations of the SINS in Pseudo-Frame

According to the SINS mechanization in Section 4.2, the pseudo-error equations can be easily derived. From Equations (15)–(18), the static PVA error equations of the SINS mechanized in pseudo-frame, which are derived in a small misalignment angles, can be described by Equations (19)–(25).

- The velocity error equations:
  (19) $$\delta {\dot{V}}_{E_{p}}=2\Omega \cos L_{p}\cos\lambda_{p}\delta V_{N_{p}}-g\phi_{y_{p}}+\nabla_{E_{p}}\;\mathrm{and}$$
  (20) $$\delta {\dot{V}}_{N_{p}}=-2\Omega \cos L_{p}\cos\lambda_{p}\delta V_{E_{p}}+g\phi_{x_{p}}+\nabla_{N_{p}}$$
- The attitude error equations:
  (21) $${\dot{\phi }}_{x_{p}}=-\Omega \cos\lambda_{p}\delta \lambda_{p}-\frac{\delta V_{N_{p}}}{R}+\Omega \cos L_{p}\cos\lambda_{p}\phi_{y_{p}}+\Omega \sin L_{p}\cos\lambda_{p}\phi_{z_{p}}+\;\varepsilon_{E_{p}}$$
  (22) $${\dot{\phi }}_{y_{p}}=-\Omega \cos L_{p}\cos\lambda_{p}\delta L_{p}+\Omega \sin L_{p}\sin\lambda_{p}\delta \lambda_{p}+\frac{\delta V_{E_{p}}}{R}-\Omega \cos L_{p}\cos\lambda_{p}\phi_{x_{p}}\;-\Omega \sin\lambda_{p}\phi_{z_{p}}+\varepsilon_{N_{p}}\;\mathrm{and}$$
  (23) $${\dot{\phi }}_{z_{p}}=-\Omega \sin L_{p}\cos\lambda_{p}\delta L_{p}-\Omega \cos L_{p}\sin\lambda_{p}\delta \lambda_{p}+\frac{\delta V_{E_{p}}}{R}\tan L_{p}-\Omega \sin L_{p}\cos\lambda_{p}\phi_{x_{p}}\;+\Omega \sin\lambda_{p}\phi_{y_{p}}+\varepsilon_{U_{p}}$$
- The position error equations:
  (24) $$\delta {\dot{L}}_{p}=\frac{\delta V_{N_{p}}}{R}\;\mathrm{and}$$
  (25) $$\delta {\dot{\lambda }}_{p}=\frac{\delta V_{E_{p}}}{R}\sec L_{p}$$

where $\nabla_{E_{p}},\nabla_{N_{p}}$ denote the accelerometer zero-biases expressed in $t_{p}$-frame; and $\varepsilon_{E_{p}},\varepsilon_{N_{p}},\varepsilon_{U_{p}}$ denote the constant gyroscope drifts expressed in $t_{p}$-frame.

## 5. The Filter Model of Kalman for Zero-Velocity Alignment

As the error equations of the SINS in the pseudo-frame have been derived in the above section, the static KF model can be given. In addition, since the alignment process of the SINS is short, the accelerometer and gyroscope errors here are regarded as random constant biases and white noise processes, namely, $\dot{\nabla }=0,\dot{\varepsilon }=0$. For the static alignment, the position errors and up velocity errors are usually ignored. Therefore, the level velocity errors, misalignment angles, the accelerometer zero-biases, and the constant gyro drifts are chosen as the state variables. The state vectors of the system error model are defined as:

$$X={\left[\begin{array}{cccccccccc} \delta V_{E_{p}} & \delta V_{N_{p}} & \phi_{x_{p}} & \phi_{y_{p}} & \phi_{z_{p}} & \nabla_{E_{p}} & \nabla_{N_{p}} & \varepsilon_{E_{p}} & \varepsilon_{N_{p}} & \varepsilon_{U_{p}} \end{array}\right]}^{T}$$

Furthermore, the state equation of static alignment for the SINS in pseudo-frame can be written in matrix form as Equation (26).

(26) $$\dot{X}(t)=F(t)X(t)+W(t)$$

where F(t) denotes the state transition matrix; and W(t) denotes the white system process noise with the power spectral density Q.

In addition, $L_{p0}$ is equal to zero in Equation (8), namely, the initial latitude in pseudo-Earth frame is zero. Therefore, referring to Equations (19)–(25), the static state transition matrix can be written as Equation (27).

(27) $$F=\left[\begin{array}{cccc} F_{11} & F_{12} & I_{2\times 2} & 0_{2\times 3}\\ F_{21} & F_{22} & 0_{3\times 2} & I_{3\times 3}\\ 0_{2\times 2} & 0_{2\times 3} & 0_{2\times 2} & 0_{2\times 3}\\ 0_{3\times 2} & 0_{3\times 3} & 0_{3\times 2} & 0_{3\times 3} \end{array}\right]$$

where

$$F_{11}=\left[\begin{array}{cc} 0 & 2\Omega \cos\lambda_{p}\\ -2\Omega \cos\lambda_{p} & 0 \end{array}\right],\;F_{12}=\left[\begin{array}{ccc} 0 & -g & 0\\ g & 0 & 0 \end{array}\right]$$

$$F_{21}=\left[\begin{array}{cc} -\frac{1}{R} & 0\\ 0 & \frac{1}{R}\\ 0 & 0 \end{array}\right],\;\mathrm{and}\;F_{22}=\left[\begin{array}{ccc} 0 & \Omega \cos\lambda_{p} & 0\\ -\Omega \cos\lambda_{p} & 0 & -\Omega \sin\lambda_{p}\\ 0 & -\Omega \sin\lambda_{p} & 0 \end{array}\right]$$

In matrix $F_{21}$, all elements in the third row are all zero. As a result, the proposed mechanization achieves the decoupling between the level linear movement errors and the heading error. It can also eliminate the influences of level linear movement errors on the heading error. Moreover, the initial latitude in pseudo-Earth frame is zero all the time. Consequently, the heading error would barely be influenced by the position (due to the mechanization). Therefore, with the innovative mechanization, the spread of the heading error influenced by the level linear movement errors (due to the latitude) can be removed without much additional complexity. For the fine alignment associated with the Kalman filter, this structural mode can eliminate the influences on the alignment performance and the loop stability from the heading error caused by the linear movement errors and the latitude, especially in the high-latitude region, including the polar region.

When the vehicle is in stationary base, the zero-velocity information in the navigation frame (namely, $t_{p}$-frame) is usually selected as the external reference. Therefore, the measurement equation can be written as Equation (28).

(28) $$Z={\left[\begin{array}{cc} V_{E_{p}}-0 & V_{N_{p}}-0 \end{array}\right]}^{T}=\left[\begin{array}{cc} \delta V_{E_{p}} & \delta V_{N_{p}} \end{array}\right]$$

where $\delta V_{E_{p}}$ and $\delta V_{N_{p}}$ are the measurement variables, which are equal to $V_{E_{p}}$ and $V_{N_{p}}$ from the SINS mechanized in pseudo-geographic frame.

Therefore, the system measurement equation can be written as Equation (29).

(29) Z(t)=H(t)X(t)+V(t)

where V(t) denotes the white measurement noise with the power spectral density R; and H(t) denotes the measurement matrix. According to Equation (28), the measurement matrix can be written as Equation (30).

(30) $$H=[\begin{array}{cc} I_{2\times 2} & 0_{2\times 8} \end{array}]$$

## 6. Simulations and Experiments

In this section, both simulations and experiments are performed to verify the performance of the static alignment with the proposed mechanization, which is assisted by the open-loop and closed-loop KFs, respectively. Firstly, under different coarse alignment accuracy conditions, the simulations of the fine alignment with the proposed algorithm and the traditional one are done. Then, the experiments are also conducted In addition, the polar alignment of the SINS mechanized in the pseudo-geographic frame is performed to demonstrate the superior performance of the proposed approach.

### 6.1. The Simulations of Fine Alignment Assisted by the KF

In this section, two groups of 300 s static data are obtained by the SINS simulator to verify the alignment performance assisted by the open-loop and closed-loop KFs, respectively. The conditions of the simulation are as follows: The initial longitude and latitude are 45° and 126°, respectively; the true attitudes of INS are all set to zero; the constant and random drifts of gyroscope are 0.01°/h and 0.001°/h, respectively; the constant and random biases of the accelerometer are 100 μg and 10 μg, respectively, the update rate is 100 Hz, and the model of the static SINS simulator is described as follows:

(31) $$\omega_{ib}=C_{n}^{b}\left[\begin{array}{c} 0\\ \Omega \cos L\\ \Omega \sin L \end{array}\right]+\left[\begin{array}{c} \varepsilon_{xcons}\\ \varepsilon_{ycons}\\ \varepsilon_{zcons} \end{array}\right]+\left[\begin{array}{c} \varepsilon_{xrandn}\\ \varepsilon_{yrandn}\\ \varepsilon_{zrandn} \end{array}\right]\;\mathrm{and}$$

(32) $$f_{ib}=C_{n}^{b}\left[\begin{array}{c} 0\\ 0\\ g_{n} \end{array}\right]+\left[\begin{array}{c} \nabla_{xcons}\\ \nabla_{ycons}\\ \nabla_{zcons} \end{array}\right]+\left[\begin{array}{c} \nabla_{xrandn}\\ \nabla_{yrandn}\\ \nabla_{zrandn} \end{array}\right]$$

where $\omega_{ib}$ and $f_{ib}$ denote outputs of gyroscopes and the accelerometers; $g_{n}$ denotes the gravity; $\varepsilon_{xcons}$, $\varepsilon_{ycons}$, $\varepsilon_{zcons}$ and $\nabla_{xcons}$, $\nabla_{ycons}$, $\nabla_{zcons}$ denote the constant drifts and the constant biases, respectively; $\varepsilon_{xrandn}$, $\varepsilon_{yrandn}$, and $\varepsilon_{zrandn}$ are white noise, and its mean is equal to random drifts; $\nabla_{xrandn}$, $\nabla_{yrandn}$, and $\nabla_{zrandn}$ are also white noise, and its mean is equal to random biases.

#### 6.1.1. The Simulations Based on the Open-Loop KF

**Figure 3:**
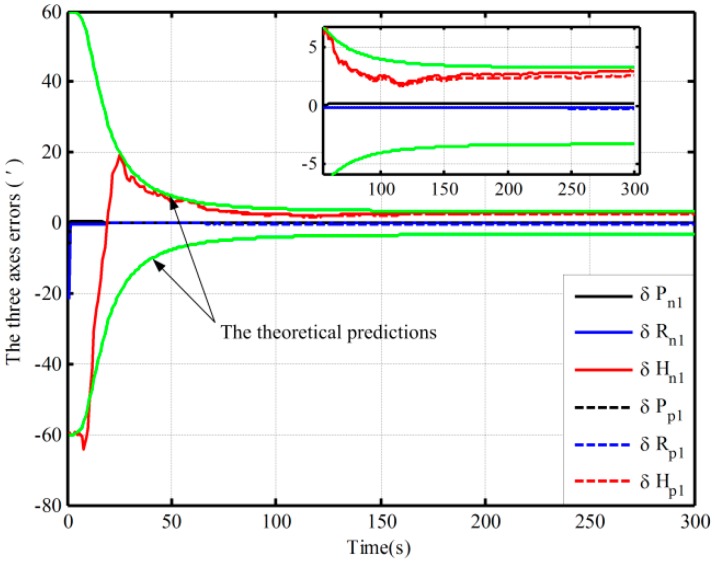
The open-loop alignment simulation results in Case 1.

**Figure 4:**
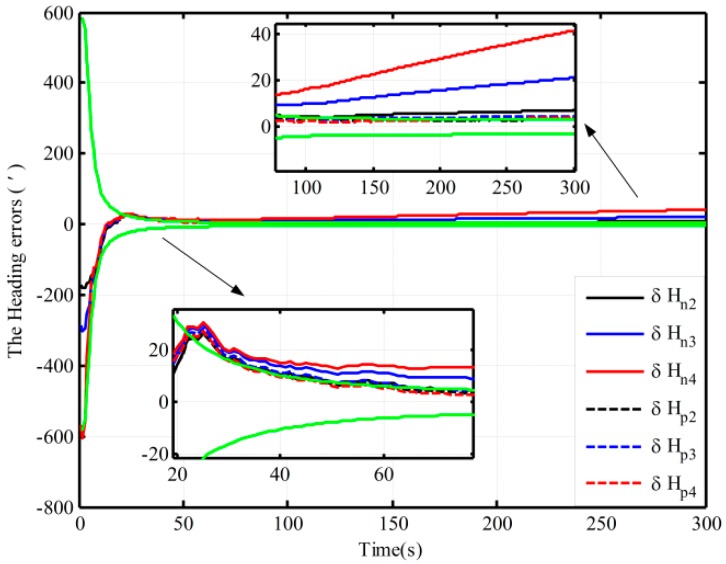
The open-loop alignment simulation results in the other three cases.

Using the proposed and the traditional mechanization, the alignment simulation assisted by open-loop KF is conducted by intentionally adding initial attitude errors. The different initial conditions of fine alignment (namely, the different coarse alignment accuracy) are shown in Table 1. Under Case 1, the alignment results (pitch, roll, and heading) based on the open-loop KF are shown in Figure 3. Due to the limitation of figures, under the other three cases, only the alignment results of the heading are shown in Figure 4.

Figure 3 shows the alignment error curves of the attitude by intentionally adding initial attitude errors (1° for heading; 0.3° for both pitch and roll), where the theoretical predictions of the heading (square root of $P_{k}$; for the matrix $P_{k}$, if not explicitly stated, all matrices $P_{k}$ denote the error variance matrix in this paper) is denoted by the green solid lines. In Figure 4, only the alignment results of the heading under the other three cases are shown, where the theoretical predictions of the heading (the square root of $P_{k}$ in Case 4) are shown with the green solid lines. In Figure 3 and Figure 4, the dotted lines represent the estimations by the proposed algorithm, solid lines denote the estimations based on the traditional algorithm, and the partial magnifications of the alignment results are also shown in the two figures.

From Figure 3, we can see that the alignment errors (pitch, roll, and heading) based on the two mechanizations are 0.19′, −0.15′, 2.75′ and 0.20′, −0.19′, 2.42′ in 200 s, respectively. The level and heading alignment results obtained by the two methods are similar, but the heading error is smaller when the proposed mechanization is used. The heading errors obtained by the two methods are also within the theoretical bounds. However, under the other three cases, the heading alignment error curves with the traditional mechanization display an obvious difference and diverge with the poor coarse alignment from Figure 4, and they are outside the theoretical bounds most of the time, indicating that the estimated effects of the open-loop KF are failed. However, with the proposed mechanization, the three estimated curves of the heading error converge over time, and the estimated results are almost the same and are all within the theoretical bounds. In addition, comparing Figure 3 with Figure 4, under the above four initial conditions, the heading estimated errors with the proposed mechanization in 200 s are 2.42′, 2.82′, 4′, and 2.98′, respectively. There is almost no difference. As a result, we conclude that the performance of the new algorithm based on the open-loop KF is superior to that of the normal one, and the proposed mechanization requires less coarse alignment accuracy to maintain the desired performance of fine alignment, whereas the traditional method requires more accurate coarse alignment. Otherwise, the estimated results of the open-loop KF will decline or even fail. Consequently, the proposed method can improve the open-loop system reliability and decrease the total alignment time because of the low requirement of coarse alignment accuracy.

#### 6.1.2. The Simulations Based on the Closed-Loop KF

**Figure 5:**
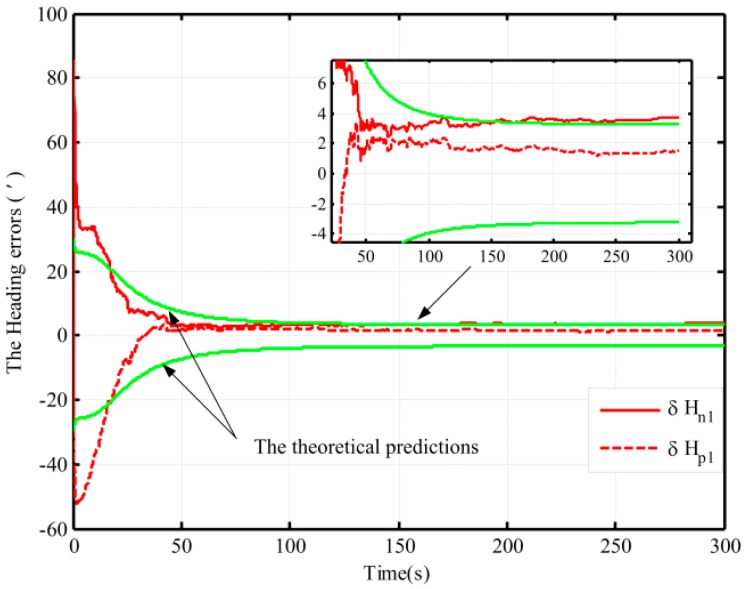
The closed-loop simulations of alignment in Case 1.

**Figure 6:**
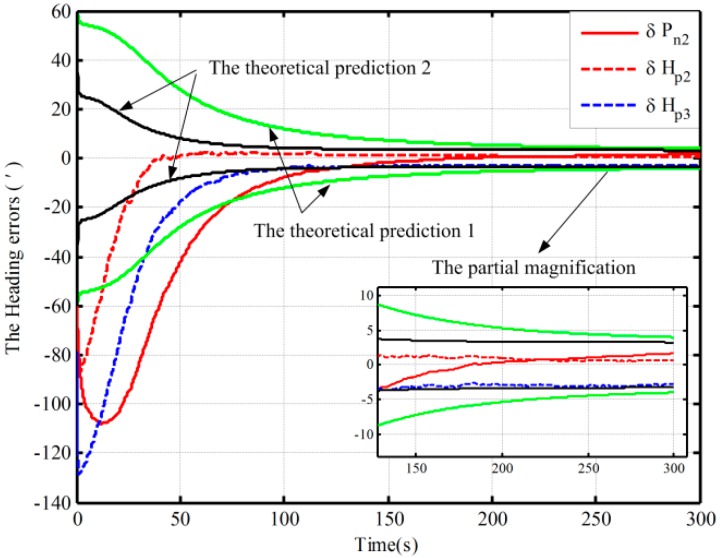
The closed-loop simulations of alignment in the other two cases.

In this section, simulations of fine assisted by the closed-loop KF are performed, and the different initial conditions of fine alignment are shown in Table 2. Under Case 1, the alignment results with the two mechanizations are shown in Figure 5. Due to divergence under the other two conditions, the alignment based on the traditional mechanization is conducted by setting larger initial values of the noise matrix to ensure the closed-loop system stability. As a result, the other two alignment results based on the proposed mechanization and the alignment result based on the traditional one in Case 2 are shown in Figure 6.

In Figure 5 and Figure 6, the dotted lines represent the estimations by the proposed algorithm and solid lines denote the estimations based on the traditional algorithm, and the partial magnifications of the alignment results are also shown. In Figure 6, the theoretical predictions of the heading (the square root of $P_{k}$ in Case 2) based on the proposed algorithm and the traditional one are denoted by a black solid line and a green solid line, respectively. The red line denotes the alignment results in Case 2, and the blue line denotes the alignment results in Case 3.

From Figure 5, under the condition of better coarse alignment (Case 1 in Table 2), the estimation result with the proposed algorithm is always within the theoretical bounds after 50 s. However, the one based on the traditional algorithm is outside the theoretical bounds after 150 s. In addition, the heading error based on the proposed mechanization is smaller, and the heading errors based on the proposed mechanization and traditional one are 1.6′ and 3.6′, respectively, in 200 s. Moreover, the estimation oscillation based on the traditional mechanization is more obvious at the beginning of the alignment, and the maximum amplitudes based on the two methods are 86′ and 52′, respectively. The violent oscillation, which may lead to the divergence of the closed-loop KF, will be very perilous. However, the proposed mechanization eliminates the effects of linear movement errors on the heading error by decoupling and hence can improve system performance. Therefore, the performance of the proposed algorithm in the closed-loop system is superior to that of the normal one.

Under the other two initial conditions, with the coarse alignment becoming poor (Cases 2 and 3 in Table 2), the alignment based on the traditional mechanization would diverge. However, from Figure 6, the proposed one is still favorable, and the alignment results in 200 s are 1.7′and −2.8′, respectively. Moreover, the estimation oscillations based on the proposed algorithm are still smaller. Therefore, the proposed mechanization requires less coarse alignment accuracy to maintain the desired performance of fine alignment, whereas the traditional method requires a higher accuracy of coarse alignment. The closed-loop estimated results would otherwise fail. Consequently, the proposed method can improve closed-loop system reliability and decrease total alignment time because of the low requirement of coarse alignment accuracy.

On the other hand, due to the fact that the closed-loop system with the traditional algorithm diverges in Case 2, a method of setting larger initial values of the noise matrix is used to maintain system stability. This alignment result is shown with a solid red line in Figure 6. The theoretical prediction 1 in Figure 6 is the square root of $P_{k}$ based on the traditional algorithm. And the square root of $P_{k}$ based on the proposed one in case 2 is denoted by the theoretical prediction 2 in figure 6. Compared with the alignment results with the two methods in Case 2 (the dotted red line and solid line), the estimation oscillation based on the traditional mechanization is still larger at the beginning. Moreover, compared with the theoretical predictions 1 and 2 in Figure 6, the alignment error of heading, which is obtained by using the traditional mechanization and setting larger initial values of the noise matrix, is larger at the end of alignment, and the speed of the convergence is obviously slower. Therefore, the closed-loop system stability can be ensured by setting larger initial values of the noise matrix, but at the expense of the alignment time and accuracy. As a result, the proposed algorithm based on the closed-loop KF has superior performance without much additional complexity.

**Figure 7:**
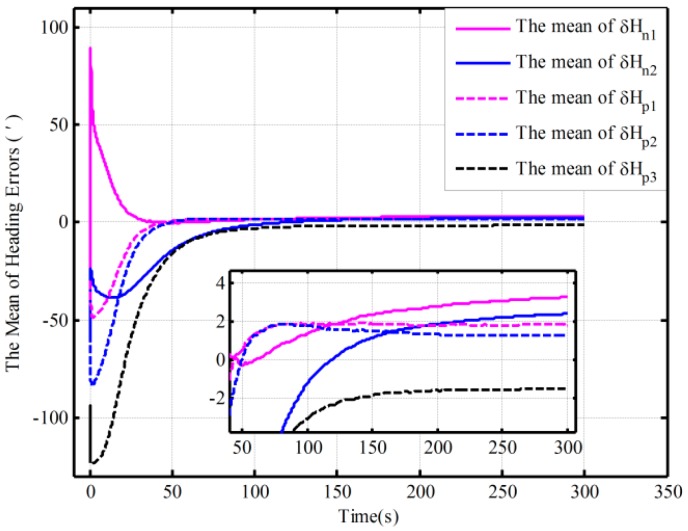
The mean error curves of heading based on the closed-loop KF.

**Figure 8:**
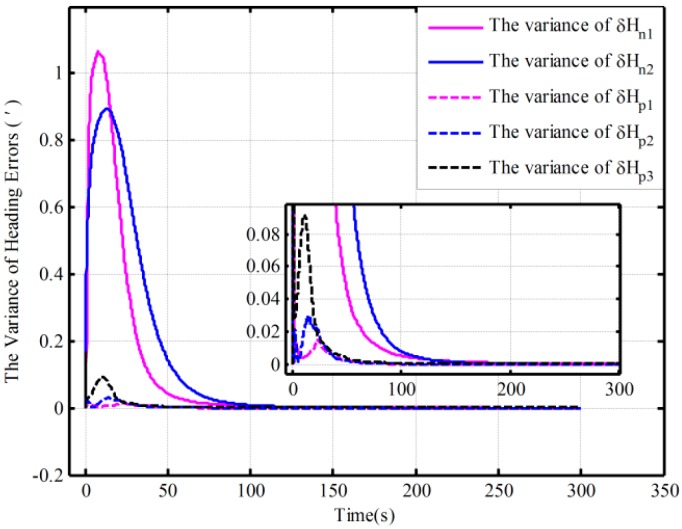
The error variance curves of heading based on the closed-loop KF.

In addition, the same behaviors assisted by the open-loop and closed-loop KFs have also been performed with the Monte Carlo simulation. Table 3 shows the statistics of the 30 heading alignment results based on the open-loop KF in 200 s. Moreover, by using the proposed algorithm, the traditional algorithm and the corrected algorithm, respectively, statistics of the 50 heading alignment results based on the closed-loop KF in 200 s are shown in Table 4, and the mean error curves and error variance curves of heading error based on the Monte Carlo simulation are shown in Figure 7 and Figure 8. From Table 3, under the above four initial conditions in Table 1, the open-loop mean errors based on the proposed algorithm are almost the same. However, the ones based on the traditional algorithm will be deteriorating with poor coarse alignment. For the closed-loop KF, the mean errors based on the proposed algorithm under the different conditions in Table 2 are also favorable from Table 4. From Figure 7 and Figure 8, for the closed-loop KF, the proposed algorithm can decrease oscillation, improve the closed-loop system stability, and reduce the requirement for coarse alignment accuracy without much additional complexity and at the expense of alignment performance. This is consistent with the results of the above analyses. Therefore, the proposed mechanization has a good capability in the SINS alignment assisted by the open-loop or closed-loop KFs.

### 6.2. The Experiments of Fine Alignment Based on the KF

**Figure 9:**
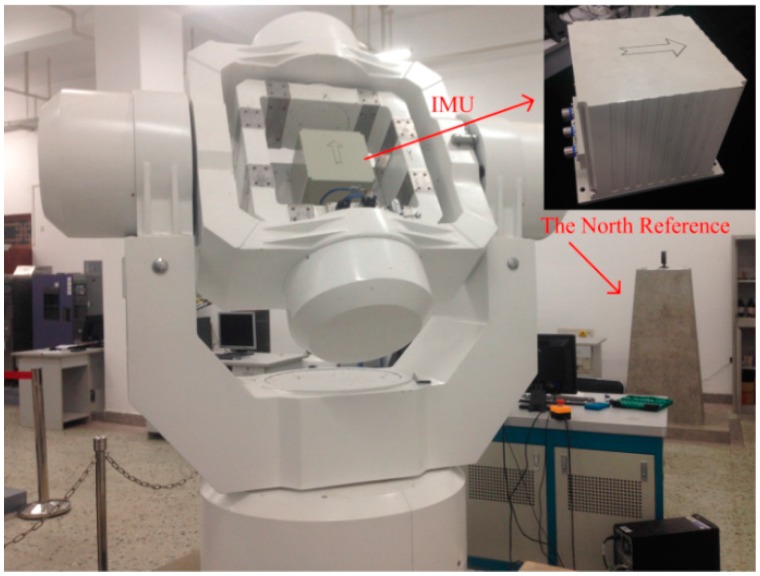
The IMU and turntable.

The experimental data were collected with the fiber optical gyroscope (FOG) SINS, fixed in a 3-axis high-precision turntable as shown in Figure 9. The inertial measurement unit (IMU) and the North Reference are also shown in the figure. The FOG SINS was made by our lab (namely, The Institute of Inertial Navigation and M&C Technology), and its sampling frequency is 100 Hz. The local longitude and latitude is 126.6778° and 45.7778°, respectively. Moreover, the attitude reference value of IMU is provided by the 3-axis high-precision turntable.

**Figure 10:**
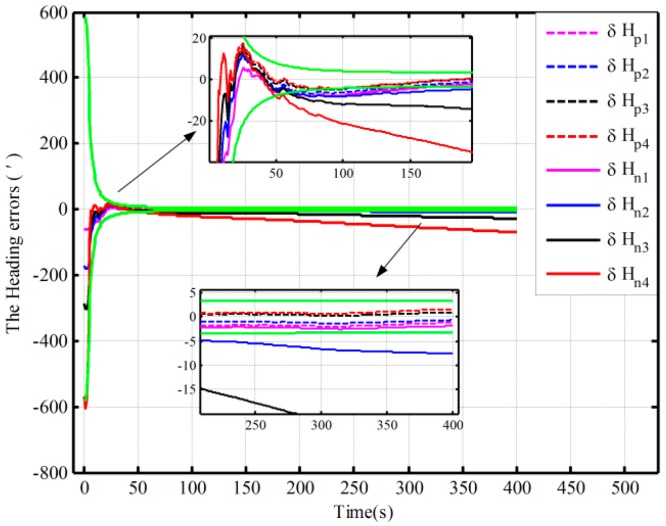
The alignment experiments based on the open-loop KF.

**Figure 11:**
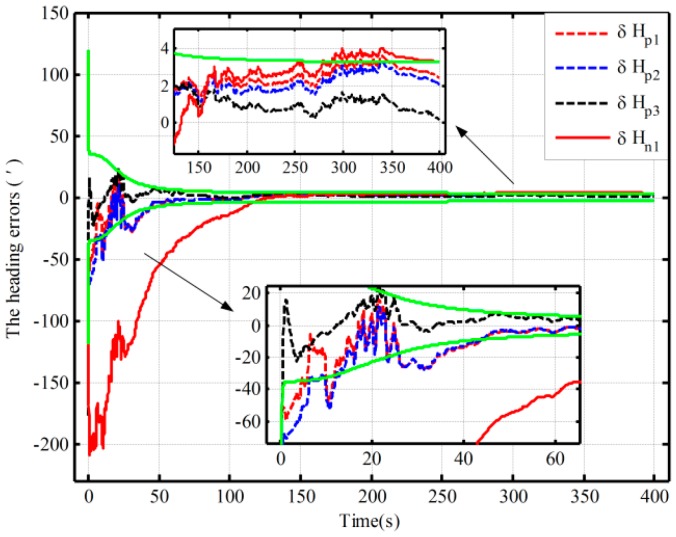
The alignment experiments based on the closed-loop KF.

**Figure 12:**
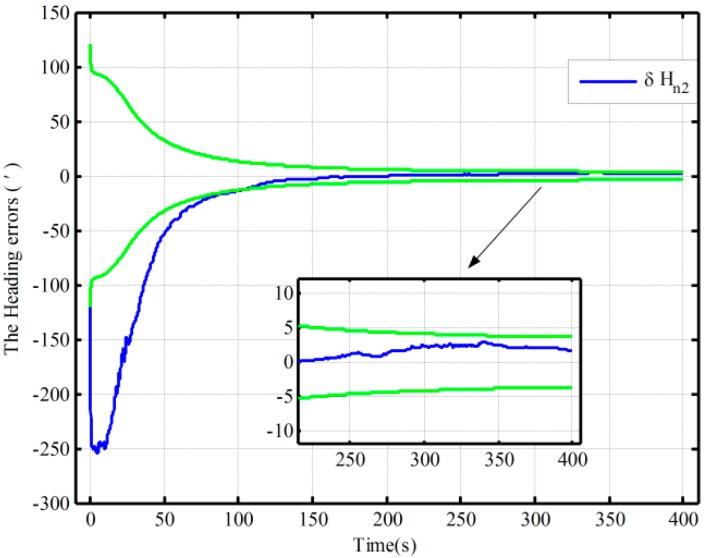
The alignment experiments with the correctional traditional algorithm in Case 2.

The fine alignment experiments based on the proposed algorithm and the traditional one are done, which is assisted by the open-loop and closed-loop KFs, respectively. The initial conditions of the open-loop KF alignment are given in Table 1, and the closed-loop KF alignment initial conditions is described in Table 5. The alignment results (only heading error) based on the open-loop KF are shown in Figure 10. Figure 11 and Figure 12 show the alignment results based on the closed-loop KF.

By using the proposed mechanization and the traditional one, the open-loop alignment error curves in the four cases in Table 1 are shown Figure 10. In addition, due to the fact that the closed-loop alignment in the traditional method diverges in Cases 2 and 3 in Table 5, the closed-loop alignment errors, only including those heading alignment errors by using the traditional method in Case 1 and the proposed method in the three cases in Table 5, are presented in Figure 11. Moreover, Figure 12 shows the closed-loop alignment error, which is obtained with the traditional method in Case 2 by setting larger initial values of the noise matrix to ensure system stability. In Figure 10, Figure 11 and Figure 12, the dotted lines represent the estimations of the proposed algorithm, and solid lines denote the estimations based on the traditional algorithm. The partial magnifications of the alignment results are also shown in the three figures, and the green solid lines denote the theoretical predictions of the heading, respectively. In Figure 11 and Figure 12, the theoretical predictions of the heading are the square root of $P_{k}$ in Case 2 by using the proposed mechanization and the traditional one with the correction, respectively.

From Figure 10, under the above four initial conditions in Table 1, the open-loop estimated heading errors with the proposed mechanization in 300 s are −1.96′, −1.32′, 0.25′, and 0.68′, respectively. There is almost no difference. However, the alignment error based on the traditional mechanization is smaller only when the coarse alignment is better (in Case 1 in Table 1). The other three alignment error curves display an obvious difference and diverge with the poor coarse alignment (in the other three cases in Table 1). Therefore, the traditional method requires more accurate coarse alignment. The open-loop estimated results would otherwise decline or even fail, whereas the proposed method can still maintain the desired performance. This is consistent with the above open-loop simulation results.

As seen from Figure 11, under the above three initial conditions in Table 5, the difference of the closed-loop alignment error curves based on the proposed method is very small, and the alignment results in 300 s are 3′, 3.4′, and 1.6′, respectively. However, the closed-loop alignment with the traditional method is convergent only when the coarse alignment is better (in Case 1 in Table 5), and the alignment result in 300 s is 4′. Compared with the alignment results with the two methods in Case 1 (the dotted red line and solid line in Figure 11), the estimation oscillation based on the traditional mechanization is very large at the beginning. However, the oscillations based on the proposed algorithm are all smaller under the conditions of the three different coarse alignments in Table 5. In addition, comparing Figure 11 with Figure 12, the closed-loop system stability based on the traditional algorithm can be ensured by setting larger initial values of the noise matrix but at the expense of alignment time and accuracy. The theoretical value and convergence time of the traditional alignment in Case 2 in Table 5 are 4.1′ and 200 s, respectively, whereas they are 3.4′ and 80 s when using the proposed alignment method. Consequently, the proposed algorithm can decrease estimation oscillation caused by system errors and improve closed-loop system stability. This is also consistent with the above closed-loop simulation results. Therefore, the proposed algorithm has superior performance in both open-loop and closed-loop KFs.

### 6.3. The Simulations of Polar Alignment

**Figure 13:**
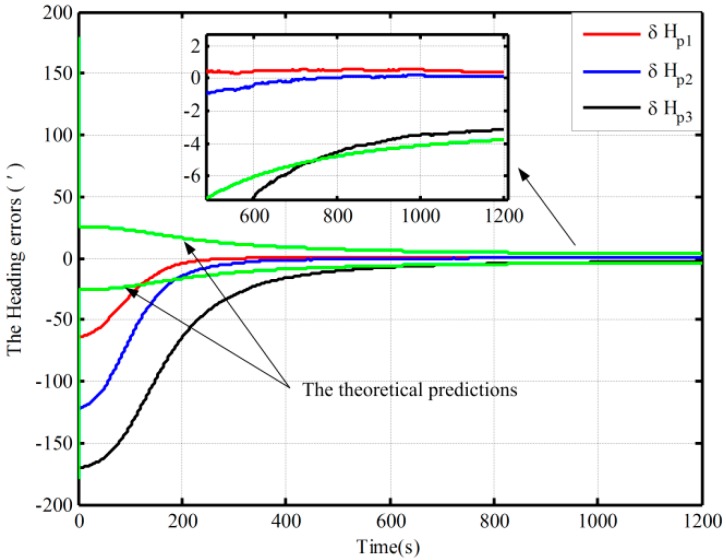
The polar closed-loop KF alignment at 85° latitude.

**Figure 14:**
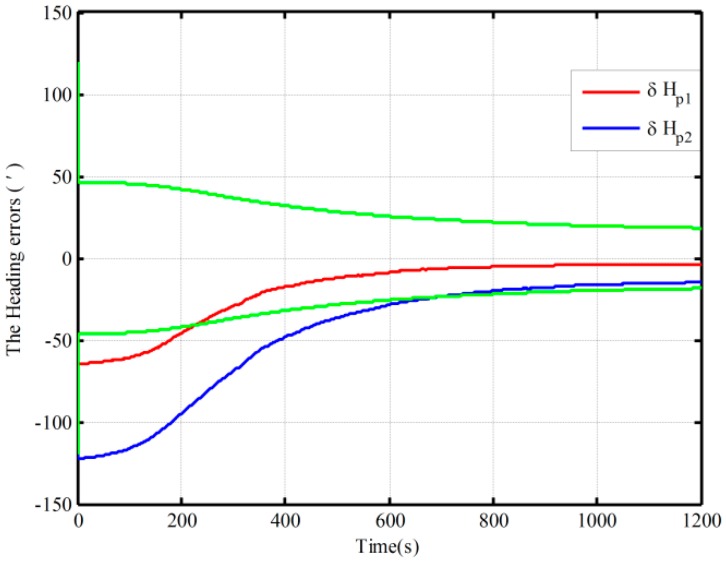
The polar closed-loop KF alignment at 89° latitude.

The simulations of polar alignment based on the proposed mechanization and the closed-loop KF were conducted. Furthermore, two groups of static data in different positions were obtained by the SINS simulator. The conditions of the simulation are as follows: The initial longitude and latitude are (126°, 85°) and (126°, 89°); the true attitudes of INS are all set to zero; the constant and random drifts of gyroscope are 0.001°/h and 0.0001°/h, respectively; and the constant and random biases of the accelerometer are 50 μg and 5 μg, respectively. At the 85° latitude the three alignment results of heading with the different initial conditions in Table 5 are shown in Figure 13. Figure 14 shows the 89° latitude alignment results in Cases 1 and 2 in Table 5.

As seen from Figure 13 and Figure 14, all the estimation curves converge with time. Figure 13 shows the polar alignment results based on the proposed mechanization under the three different initial conditions when the latitude is 85°, where the heading alignment errors are all less than 3.8′ in 1200 s. The heading alignment errors under the two cases are less than 18′ in 1200 s at 89° latitudes from Figure 14, and the alignment results do not display an obvious difference under different initial conditions in the two figures. On the other hand, the highly accurate coarse alignment is also difficult to achieve due to the smaller gyrocompass component. As a result, the proposed mechanization has a good ability in polar alignment because it requires less accurate coarse alignment. Therefore, the proposed mechanization can solve the problem of polar alignment coming from the coupling between the level movement errors and the heading error.

Furthermore, comparing Figure 13 with Figure 14, the convergence speed at 89° is slower than that at 85°, and the alignment accuracy is also lower than the one at 85°. This is consistent with the problem arising in polar self-alignment. The decline of gyrocompass component leads to a slow convergence speed and poor alignment accuracy in the polar region. Therefore, self-alignment will fail in poles or near the poles. Finding other solutions to achieve the alignment in the poles or near the poles will be pursued in future work.

## 7. Conclusions

The main objective of this paper is to solve the problems of KF alignment and polar alignment on stationary base. An innovative mechanization is proposed to achieve fine alignment for the SINS. The improved mechanization eliminates the effects of linear movement errors and latitude on the heading error by decoupling. Compared with the traditional mechanization, the proposed algorithm has superior performance without much additional complexity. The proposed algorithm would require lower coarse alignment accuracy in both the open-loop and closed-loop KFs and hence can improve the system reliability and decrease total alignment time. For the closed-loop KF, it can also decrease estimation oscillation caused by system errors and improve closed-loop system stability. Moreover, the proposed algorithm can also be applied to polar alignment. Finally, the simulations and experiments are conducted, and the results exhibit the superior performance of the proposed approach. As a result, the proposed algorithm is promising for polar alignment and KF alignment. On the other hand, though motivated by the SINS static alignment, it can be applied to quasi-static alignment and PINS alignment. For the PINS, only a switching control is required from the alignment stage to the navigation stage. In addition, as the proposed mechanization is similar to the traditional local-level north mechanization, the new method has a high project value.

## Figures and Tables

**Table 1:** The different initial conditions for the open-loop KF.

The Coarse Alignment Accuracy	Errors		
	Pitch	Roll	Heading
Case 1	0.3°	0.3°	1°
Case 2	1°	1°	3°
Case 3	2°	2°	5°
Case 4	3°	3°	10°

**Table 2:** The different initial conditions for the closed-loop simulations.

The Coarse Alignment Accuracy	Errors		
	Pitch	Roll	Heading
Case 1	0.2°	0.2°	0.5°
Case 2	0.3°	0.3°	1°
Case 3	0.5°	0.5°	2°

**Table 3:** Statistics of heading errors based on the open-loop Kalman filter (KF).

Conditions	The Proposed Algorithm		The Traditional Algorithm	
	Mean	Variance	Mean	Variance
Case 1	1.57′	5 × 10^−4^	1.76′	6.25 × 10^−4^
Case2	1.74′	6.6 × 10^−4^	3.59′	2.6 × 10^−3^
Case3	2.53′	1.28 × 10^−3^	15.9′	1.28 × 10^−5^
Case4	1.82′	7.26 × 10^−4^	28.32′	8.35 × 10^−3^

**Table 4:** Statistics of heading errors based on the closed-loop KF.

Conditions	The Proposed Algorithm		The Traditional Algorithm		The Larger Noise Matrix Algorithm	
	Mean	Variance	Mean	Variance	Mean	Variance
Case 1	1.81′	5 × 10^−5^	2.80′	8.93 × 10^−4^	−	−
Case 2	1.33′	1 × 10^−4^	×	×	1.87′	4.2 × 10^−4^
Case 3	−1.62′	1 × 10^−3^	×	×	−	−

^1.^ “×”denotes that alignment result diverges, and “−”denotes the experiment dose was not conducted.

**Table 5:** The different initial conditions for fine alignment.

The Coarse Alignment Accuracy	Errors		
	Pitch	Roll	Heading
Case 1	0.3°	0.3°	1°
Case 2	0.5°	0.5°	2°
Case 3	1°	1°	3°
